# Chlorido(2-{1-[(2-morpholino­eth­yl)imino]­eth­yl}phenolato-κ^3^ *N*,*N*′,*O*)copper(II)

**DOI:** 10.1107/S1600536810051160

**Published:** 2010-12-11

**Authors:** Nurul Azimah Ikmal Hisham, Nura Suleiman Gwaram, Hamid Khaledi, Hapipah Mohd Ali

**Affiliations:** a Department of Chemistry, University of Malaya, 50603 Kuala Lumpur, Malaysia

## Abstract

In the title compound, [CuCl(C_14_H_19_N_2_O_2_)], the Cu^II^ ion is four-coordinated by one deprotonated *N*,*N*′,*O*-tridentate Schiff base and one chloride ion in a distorted square-planar geometry. In the crystal, adjacent mol­ecules are linked *via* C—H⋯Cl and C—H⋯O inter­actions, forming infinite layers parallel to the (100) plane. The structure was determined from a non-merohedrally twined crystal [twin ratio 0.777 (3):0.223 (3)].

## Related literature

For the crystal structures of similar Cu^II^ complexes, see: Elias *et al.* (1982 ▶); Ikmal Hisham *et al.* (2009 ▶); Wang & You (2007 ▶).
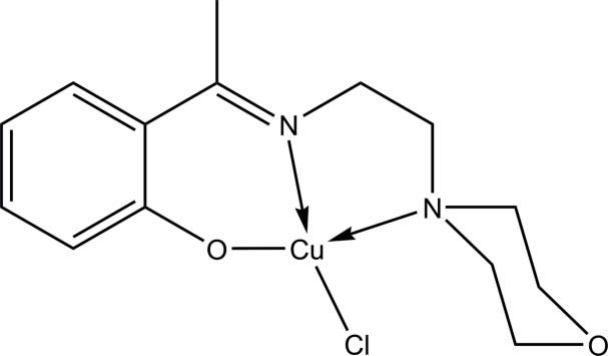


## Experimental

### 

#### Crystal data

[CuCl(C_14_H_19_N_2_O_2_)]
*M* *_r_* = 346.30
Monoclinic, 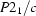

*a* = 10.7122 (4) Å
*b* = 17.1657 (7) Å
*c* = 7.7638 (3) Å
β = 93.493 (3)°
*V* = 1424.97 (10) Å^3^
*Z* = 4
Mo *K*α radiation
μ = 1.72 mm^−1^
*T* = 100 K
0.31 × 0.22 × 0.07 mm

#### Data collection

Bruker APEXII CCD diffractometer
Absorption correction: multi-scan (*SADABS*; Sheldrick, 1996 ▶) *T*_min_ = 0.617, *T*_max_ = 0.889
10834 measured reflections
2506 independent reflections
2313 reflections with *I* > 2σ(*I*)
*R*_int_ = 0.034

#### Refinement

*R*[*F* ^2^ > 2σ(*F* ^2^)] = 0.043
*wR*(*F* ^2^) = 0.096
*S* = 1.10
2506 reflections
183 parameters
H-atom parameters constrained
Δρ_max_ = 0.81 e Å^−3^
Δρ_min_ = −1.15 e Å^−3^

### 

Data collection: *APEX2* (Bruker, 2007 ▶); cell refinement: *SAINT* (Bruker, 2007 ▶); data reduction: *SAINT*; program(s) used to solve structure: *SHELXS97* (Sheldrick, 2008 ▶); program(s) used to refine structure: *SHELXL97* (Sheldrick, 2008 ▶); molecular graphics: *X-SEED* (Barbour, 2001 ▶); software used to prepare material for publication: *SHELXL97* and *publCIF* (Westrip, 2010 ▶).

## Supplementary Material

Crystal structure: contains datablocks I, global. DOI: 10.1107/S1600536810051160/gk2329sup1.cif

Structure factors: contains datablocks I. DOI: 10.1107/S1600536810051160/gk2329Isup2.hkl

Additional supplementary materials:  crystallographic information; 3D view; checkCIF report

## Figures and Tables

**Table:** 

Cu1—O1	1.877 (3)
Cu1—N1	1.932 (3)
Cu1—N2	2.050 (3)
Cu1—Cl1	2.2565 (11)

**Table:** 

O1—Cu1—N1	92.21 (14)
O1—Cu1—N2	162.15 (13)
N1—Cu1—N2	87.14 (14)
O1—Cu1—Cl1	92.57 (10)
N1—Cu1—Cl1	158.07 (11)
N2—Cu1—Cl1	94.66 (10)

**Table 2:** Hydrogen-bond geometry (Å, °)

*D*—H⋯*A*	*D*—H	H⋯*A*	*D*⋯*A*	*D*—H⋯*A*
C14—H14*A*⋯Cl1	0.99	2.75	3.386 (4)	123
C11—H11*B*⋯Cl1	0.99	2.78	3.409 (4)	122
C14—H14*B*⋯Cl1^i^	0.99	2.77	3.713 (4)	159
C10—H10*B*⋯O1^i^	0.99	2.52	3.465 (5)	160
C9—H9*B*⋯Cl1^ii^	0.99	2.83	3.680 (5)	144

Symmetry codes: (i) 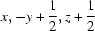; (ii) 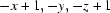.

**Fig. 1.**
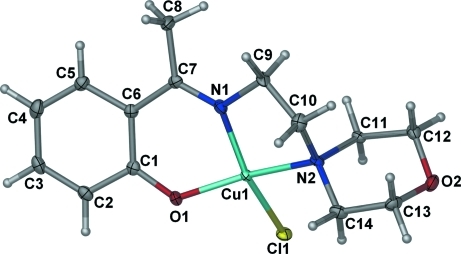
Displacement ellipsoid plot of the title compound at the 50% probability level. Hydrogen atoms are drawn as spheres of arbitrary radius.

**Fig. 2.**
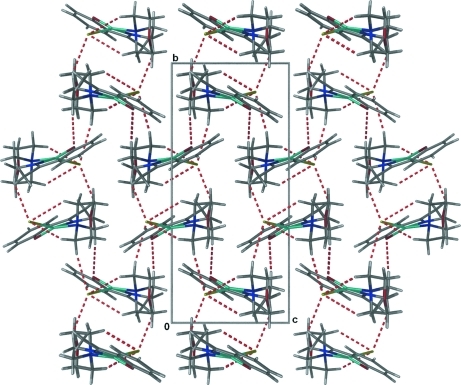
The crystal packing of the title compound down the crystallographic a axis.

## supplementary crystallographic information

### Comment

The title compound was obtained through the reaction of the Schiff base ligand, prepared *in situ*, with copper(II) chloride. Upon complexation, the Schiff base loses its phenolic hydrogen to chelate the Cu^II^ ion as an anionic tridentate ligand. One chloride atom completes the distorted square-planar geometry of the complex. The deviation from the regular geometry is evident from the disposition of the metal atom 0.0494 (15) Å out of the N1—N2—O1—Cl1 coordination plane. The Cu—N, Cu—O and Cu—Cl bond lengths in the present complex are comparable with those in similar structures [Elias *et al.*, 1982; Ikmal Hisham *et al.*, 2009; Wang & You, 2007]. In the crystal, C—H···Cl and C—H···O interactions within the range for normal hydrogen bonds, link adjacent molecules into two-dimensional networks parallel to the *bc* plane (Fig. 2). In addition, intramolecular C—H···Cl hydrogen bonds occurs.

### Experimental

A mixture of 2-hydroxyacetophenone (0.5 g, 3.7 mmol) and 4-(2-aminoethyl)morpholine (0.48 g, 3.7 mmol) in ethanol (20 ml) was refluxed for 2 hr followed by addition of a solution of copper(II) chloride dihydrate (0.63 g, 3.7 mmol) in a minimum amount of ethanol. The resulting solution was refluxed for 30 min, then left at room temperature. The crystals of the title complex were obtained after a few days.

### Refinement

The hydrogen atoms were placed at calculated positions (C—H 0.95 - 0.99 Å) and were treated as riding on their parent atoms with *U_iso_*(H) set to 1.2–1.5 *Ueq*(C).The structure was a determined from a non-merohedrally twinned specimen;  twin law in reciprocal space 1 0 0.168  0 - 1 0 0 0 - 1; SHELXL-97 (Sheldrick, 2008) BASF parameter 0.223 (3).

### Crystal data

**Table:**

[CuCl(C_14_H_19_N_2_O_2_)]	*F*(000) = 716
*M**_r_* = 346.30	*D*_x_ = 1.614 Mg m^−^^3^
Monoclinic, *P*2_1_/*c*	Mo *K*α radiation, λ = 0.71073 Å
Hall symbol: -P 2ybc	Cell parameters from 4663 reflections
*a* = 10.7122 (4) Å	θ = 2.4–28.8°
*b* = 17.1657 (7) Å	µ = 1.72 mm^−^^1^
*c* = 7.7638 (3) Å	*T* = 100 K
β = 93.493 (3)°	Plate, blue
*V* = 1424.97 (10) Å^3^	0.31 × 0.22 × 0.07 mm
*Z* = 4	

### Data collection

**Table:**

Bruker APEXII CCD diffractometer	2506 independent reflections
Radiation source: fine-focus sealed tube	2313 reflections with *I* > 2σ(*I*)
graphite	*R*_int_ = 0.034
φ and ω scans	θ_max_ = 25.0°, θ_min_ = 2.2°
Absorption correction: multi-scan (*SADABS*; Sheldrick, 1996)	*h* = −12→12
*T*_min_ = 0.617, *T*_max_ = 0.889	*k* = −20→20
10834 measured reflections	*l* = −6→9

### Refinement

**Table:**

Refinement on *F*^2^	Primary atom site location: structure-invariant direct methods
Least-squares matrix: full	Secondary atom site location: difference Fourier map
*R*[*F*^2^ > 2σ(*F*^2^)] = 0.043	Hydrogen site location: inferred from neighbouring sites
*wR*(*F*^2^) = 0.096	H-atom parameters constrained
*S* = 1.10	*w* = 1/[σ^2^(*F*_o_^2^) + (0.*P*)^2^ + 6.7834*P*] where *P* = (*F*_o_^2^ + 2*F*_c_^2^)/3
2506 reflections	(Δ/σ)_max_ < 0.001
183 parameters	Δρ_max_ = 0.81 e Å^−^^3^
0 restraints	Δρ_min_ = −1.15 e Å^−^^3^

### Special details

**Table:**

Geometry. All e.s.d.'s (except the e.s.d. in the dihedral angle between two l.s. planes) are estimated using the full covariance matrix. The cell e.s.d.'s are taken into account individually in the estimation of e.s.d.'s in distances, angles and torsion angles; correlations between e.s.d.'s in cell parameters are only used when they are defined by crystal symmetry. An approximate (isotropic) treatment of cell e.s.d.'s is used for estimating e.s.d.'s involving l.s. planes.
Refinement. Refinement of *F*^2^ against ALL reflections. The weighted *R*-factor *wR* and goodness of fit *S* are based on *F*^2^, conventional *R*-factors *R* are based on *F*, with *F* set to zero for negative *F*^2^. The threshold expression of *F*^2^ > σ(*F*^2^) is used only for calculating *R*-factors(gt) *etc*. and is not relevant to the choice of reflections for refinement. *R*-factors based on *F*^2^ are statistically about twice as large as those based on *F*, and *R*- factors based on ALL data will be even larger.

### Fractional atomic coordinates and isotropic or equivalent isotropic displacement parameters (Å^2^)

**Table:**

	*x*	*y*	*z*	*U*_iso_*/*U*_eq_	
Cu1	0.51091 (4)	0.13385 (3)	0.49248 (6)	0.01131 (15)	
Cl1	0.35700 (9)	0.11054 (6)	0.28748 (12)	0.0166 (2)	
O1	0.6167 (3)	0.17572 (17)	0.3321 (4)	0.0177 (6)	
O2	0.1491 (3)	0.09423 (18)	0.8080 (4)	0.0209 (7)	
N1	0.6497 (3)	0.1116 (2)	0.6556 (4)	0.0152 (8)	
N2	0.3974 (3)	0.12315 (19)	0.6943 (4)	0.0125 (7)	
C1	0.7370 (4)	0.1654 (2)	0.3291 (5)	0.0138 (8)	
C2	0.7957 (4)	0.1964 (2)	0.1849 (6)	0.0169 (9)	
H2	0.7470	0.2253	0.1008	0.020*	
C3	0.9208 (4)	0.1858 (2)	0.1639 (6)	0.0195 (10)	
H3	0.9571	0.2063	0.0648	0.023*	
C4	0.9953 (4)	0.1448 (3)	0.2879 (6)	0.0224 (10)	
H4	1.0816	0.1365	0.2722	0.027*	
C5	0.9423 (4)	0.1168 (2)	0.4322 (6)	0.0168 (9)	
H5	0.9939	0.0903	0.5173	0.020*	
C6	0.8129 (4)	0.1261 (2)	0.4589 (5)	0.0140 (8)	
C7	0.7669 (4)	0.1010 (2)	0.6241 (6)	0.0141 (9)	
C8	0.8561 (4)	0.0640 (3)	0.7551 (6)	0.0217 (10)	
H8A	0.8092	0.0353	0.8391	0.033*	
H8B	0.9068	0.1044	0.8148	0.033*	
H8C	0.9110	0.0279	0.6974	0.033*	
C9	0.6058 (4)	0.0955 (3)	0.8300 (5)	0.0171 (9)	
H9A	0.6696	0.1123	0.9199	0.021*	
H9B	0.5911	0.0389	0.8440	0.021*	
C10	0.4853 (4)	0.1402 (3)	0.8475 (6)	0.0206 (9)	
H10A	0.4467	0.1245	0.9549	0.025*	
H10B	0.5030	0.1967	0.8539	0.025*	
C11	0.3417 (4)	0.0438 (2)	0.6993 (5)	0.0122 (8)	
H11A	0.4089	0.0052	0.7246	0.015*	
H11B	0.3010	0.0313	0.5847	0.015*	
C12	0.2454 (4)	0.0379 (2)	0.8359 (5)	0.0170 (9)	
H12A	0.2082	−0.0149	0.8327	0.020*	
H12B	0.2875	0.0457	0.9517	0.020*	
C13	0.2030 (4)	0.1704 (3)	0.8178 (6)	0.0204 (10)	
H13A	0.2462	0.1780	0.9329	0.025*	
H13B	0.1359	0.2100	0.8033	0.025*	
C14	0.2951 (4)	0.1817 (2)	0.6801 (6)	0.0187 (9)	
H14A	0.2506	0.1775	0.5649	0.022*	
H14B	0.3315	0.2346	0.6906	0.022*	

### Atomic displacement parameters (Å^2^)

**Table:**

	*U*^11^	*U*^22^	*U*^33^	*U*^12^	*U*^13^	*U*^23^
Cu1	0.0114 (3)	0.0124 (2)	0.0102 (2)	−0.0004 (2)	0.00097 (19)	0.00130 (19)
Cl1	0.0189 (5)	0.0203 (5)	0.0101 (5)	−0.0007 (4)	−0.0027 (4)	0.0009 (4)
O1	0.0153 (15)	0.0196 (16)	0.0184 (16)	−0.0014 (12)	0.0031 (12)	0.0058 (13)
O2	0.0119 (15)	0.0287 (17)	0.0220 (17)	0.0000 (13)	0.0014 (13)	0.0008 (14)
N1	0.0170 (18)	0.0164 (18)	0.0122 (18)	−0.0036 (14)	0.0004 (15)	0.0011 (14)
N2	0.0171 (17)	0.0128 (17)	0.0073 (17)	−0.0002 (14)	−0.0006 (13)	0.0012 (13)
C1	0.015 (2)	0.0110 (19)	0.015 (2)	−0.0025 (16)	0.0002 (16)	−0.0040 (16)
C2	0.022 (2)	0.012 (2)	0.017 (2)	−0.0015 (17)	0.0020 (18)	0.0000 (17)
C3	0.025 (2)	0.016 (2)	0.018 (2)	−0.0056 (18)	0.0070 (18)	−0.0009 (18)
C4	0.016 (2)	0.020 (2)	0.032 (3)	−0.0030 (18)	0.0042 (19)	−0.004 (2)
C5	0.015 (2)	0.014 (2)	0.021 (2)	0.0006 (16)	0.0000 (18)	−0.0024 (17)
C6	0.016 (2)	0.011 (2)	0.015 (2)	0.0001 (16)	0.0013 (17)	−0.0023 (16)
C7	0.015 (2)	0.0091 (19)	0.018 (2)	−0.0009 (16)	−0.0015 (17)	−0.0016 (16)
C8	0.013 (2)	0.031 (3)	0.021 (2)	0.0014 (19)	0.0002 (18)	0.006 (2)
C9	0.018 (2)	0.026 (2)	0.007 (2)	−0.0061 (18)	−0.0030 (17)	0.0033 (17)
C10	0.020 (2)	0.022 (2)	0.020 (2)	−0.0029 (19)	0.0016 (19)	−0.0059 (19)
C11	0.017 (2)	0.0096 (19)	0.010 (2)	−0.0004 (16)	0.0003 (16)	0.0004 (15)
C12	0.018 (2)	0.020 (2)	0.014 (2)	−0.0048 (17)	−0.0005 (17)	0.0006 (17)
C13	0.017 (2)	0.022 (2)	0.022 (2)	0.0066 (18)	0.0015 (18)	−0.0004 (19)
C14	0.026 (2)	0.013 (2)	0.016 (2)	0.0030 (18)	0.0002 (18)	0.0008 (17)

### Geometric parameters (Å, °)

**Table:**

Cu1—O1	1.877 (3)	C5—H5	0.9500
Cu1—N1	1.932 (3)	C6—C7	1.466 (6)
Cu1—N2	2.050 (3)	C7—C8	1.495 (6)
Cu1—Cl1	2.2565 (11)	C8—H8A	0.9800
O1—C1	1.302 (5)	C8—H8B	0.9800
O2—C12	1.422 (5)	C8—H8C	0.9800
O2—C13	1.430 (5)	C9—C10	1.515 (6)
N1—C7	1.306 (5)	C9—H9A	0.9900
N1—C9	1.486 (5)	C9—H9B	0.9900
N2—C14	1.485 (5)	C10—H10A	0.9900
N2—C11	1.488 (5)	C10—H10B	0.9900
N2—C10	1.500 (5)	C11—C12	1.527 (6)
C1—C2	1.421 (6)	C11—H11A	0.9900
C1—C6	1.426 (6)	C11—H11B	0.9900
C2—C3	1.373 (6)	C12—H12A	0.9900
C2—H2	0.9500	C12—H12B	0.9900
C3—C4	1.401 (6)	C13—C14	1.511 (6)
C3—H3	0.9500	C13—H13A	0.9900
C4—C5	1.374 (6)	C13—H13B	0.9900
C4—H4	0.9500	C14—H14A	0.9900
C5—C6	1.423 (6)	C14—H14B	0.9900
			
O1—Cu1—N1	92.21 (14)	H8A—C8—H8B	109.5
O1—Cu1—N2	162.15 (13)	C7—C8—H8C	109.5
N1—Cu1—N2	87.14 (14)	H8A—C8—H8C	109.5
O1—Cu1—Cl1	92.57 (10)	H8B—C8—H8C	109.5
N1—Cu1—Cl1	158.07 (11)	N1—C9—C10	107.8 (3)
N2—Cu1—Cl1	94.66 (10)	N1—C9—H9A	110.1
C1—O1—Cu1	126.7 (3)	C10—C9—H9A	110.1
C12—O2—C13	109.1 (3)	N1—C9—H9B	110.1
C7—N1—C9	120.4 (4)	C10—C9—H9B	110.1
C7—N1—Cu1	127.9 (3)	H9A—C9—H9B	108.5
C9—N1—Cu1	111.1 (3)	N2—C10—C9	109.1 (3)
C14—N2—C11	109.0 (3)	N2—C10—H10A	109.9
C14—N2—C10	110.6 (3)	C9—C10—H10A	109.9
C11—N2—C10	113.0 (3)	N2—C10—H10B	109.9
C14—N2—Cu1	110.6 (2)	C9—C10—H10B	109.9
C11—N2—Cu1	111.1 (2)	H10A—C10—H10B	108.3
C10—N2—Cu1	102.4 (2)	N2—C11—C12	111.6 (3)
O1—C1—C2	116.7 (4)	N2—C11—H11A	109.3
O1—C1—C6	125.1 (4)	C12—C11—H11A	109.3
C2—C1—C6	118.2 (4)	N2—C11—H11B	109.3
C3—C2—C1	121.8 (4)	C12—C11—H11B	109.3
C3—C2—H2	119.1	H11A—C11—H11B	108.0
C1—C2—H2	119.1	O2—C12—C11	111.3 (3)
C2—C3—C4	120.3 (4)	O2—C12—H12A	109.4
C2—C3—H3	119.9	C11—C12—H12A	109.4
C4—C3—H3	119.9	O2—C12—H12B	109.4
C5—C4—C3	119.3 (4)	C11—C12—H12B	109.4
C5—C4—H4	120.3	H12A—C12—H12B	108.0
C3—C4—H4	120.3	O2—C13—C14	111.0 (4)
C4—C5—C6	122.3 (4)	O2—C13—H13A	109.4
C4—C5—H5	118.9	C14—C13—H13A	109.4
C6—C5—H5	118.9	O2—C13—H13B	109.4
C5—C6—C1	118.0 (4)	C14—C13—H13B	109.4
C5—C6—C7	118.5 (4)	H13A—C13—H13B	108.0
C1—C6—C7	123.3 (4)	N2—C14—C13	111.8 (3)
N1—C7—C6	120.1 (4)	N2—C14—H14A	109.3
N1—C7—C8	120.9 (4)	C13—C14—H14A	109.3
C6—C7—C8	119.0 (4)	N2—C14—H14B	109.3
C7—C8—H8A	109.5	C13—C14—H14B	109.3
C7—C8—H8B	109.5	H14A—C14—H14B	107.9

### Hydrogen-bond geometry (Å, °)

**Table:**

*D*—H···*A*	*D*—H	H···*A*	*D*···*A*	*D*—H···*A*
C14—H14A···Cl1	0.99	2.75	3.386 (4)	123
C11—H11B···Cl1	0.99	2.78	3.409 (4)	122
C14—H14B···Cl1^i^	0.99	2.77	3.713 (4)	159
C10—H10B···O1^i^	0.99	2.52	3.465 (5)	160
C9—H9B···Cl1^ii^	0.99	2.83	3.680 (5)	144

Symmetry codes: (i) *x*, −*y*+1/2, *z*+1/2; (ii) −*x*+1, −*y*, −*z*+1.
